# Quantification of Age-Dependent Somatic CAG Repeat Instability in *Hdh* CAG Knock-In Mice Reveals Different Expansion Dynamics in Striatum and Liver

**DOI:** 10.1371/journal.pone.0023647

**Published:** 2011-08-29

**Authors:** Jong-Min Lee, Ricardo Mouro Pinto, Tammy Gillis, Jason C. St. Claire, Vanessa C. Wheeler

**Affiliations:** Center for Human Genetic Research, Massachusetts General Hospital, Boston, Massachusetts, United States of America; University Medical Center Groningen, The Netherlands

## Abstract

**Background:**

Age at onset of Huntington's disease (HD) is largely determined by the CAG trinucleotide repeat length in the *HTT* gene. Importantly, the CAG repeat undergoes tissue-specific somatic instability, prevalent in brain regions that are disease targets, suggesting a potential role for somatic CAG repeat instability in modifying HD pathogenesis. Thus, understanding underlying mechanisms of somatic CAG repeat instability may lead to discoveries of novel therapeutics for HD. Investigation of the dynamics of the CAG repeat size changes over time may provide insights into the mechanisms underlying CAG repeat instability.

**Methodology/Principal Findings:**

To understand how the *HTT* CAG repeat length changes over time, we quantified somatic instability of the CAG repeat in Huntington's disease CAG knock-in mice from 2–16 months of age in liver, striatum, spleen and tail. The *HTT* CAG repeat in spleen and tail was very stable, but that in liver and striatum expanded over time at an average rate of one CAG per month. Interestingly, the patterns of repeat instability were different between liver and striatum. Unstable CAG repeats in liver repeatedly gained similar sizes of additional CAG repeats (approximately two CAGs per month), maintaining a distinct population of unstable repeats. In contrast, unstable CAG repeats in striatum gained additional repeats with different sizes resulting in broadly distributed unstable CAG repeats. Expanded CAG repeats in the liver were highly enriched in polyploid hepatocytes, suggesting that the pattern of liver instability may reflect the restriction of the unstable repeats to a unique cell type.

**Conclusions/Significance:**

Our results are consistent with repeat expansion occurring as a consequence of recurrent small repeat insertions that differ in different tissues. Investigation of the specific mechanisms that underlie liver and striatal instability will contribute to our understanding of the relationship between instability and disease and the means to intervene in this process.

## Introduction

Huntington's disease (HD) is a dominantly inherited neurodegenerative disease, presenting symptoms such as involuntary movements, cognitive decline and psychiatric disturbances [1], [2]. Expansion of a CAG trinucleotide repeat in the *HTT* gene over 35 repeats causes HD [3], with the length of the expanded CAG repeat inversely correlated with age at onset of HD symptoms [4], [5], [6]. Importantly, expanded CAG repeats undergo further expansion-biased somatic instability in a tissue-specific manner, with striatum and cortex displaying the longest repeat lengths [7]. In accurate genetic HD CAG knock-in mouse models (*Hdh* CAG knock-in mice), striatum and liver show the greatest levels of repeat expansion [8], [9], [10]. Since striatum is a common target of early HD pathogenesis and CAG repeat instability, there is debate as to whether or not trinucleotide repeat-associated pathogenesis itself contributes to repeat instability. For example, by overexpressing pathogenic-length polyQ protein in flies, Jung and coworkers showed that pathogenic polyQ protein modulates repeat instability [11]. In contrast, using accurate genetic models of HD, we demonstrated that CAG repeat instability is not a consequence of HD pathogenesis, but is contributed to by multiple intrinsic tissue factors [12]. Regardless, data from mice [13], [14] and humans [15] suggest that somatic CAG instability in target cells contributes to HD pathogenesis. Therefore, understanding mechanisms of CAG repeat instability is important as this could lead to discoveries of modifiers of HD.

Another important characteristic of somatic *HTT* CAG repeat instability is its accumulation over time [8], [9], [10], [16]. Understanding the dynamics of somatic instability over time is important as this could provide insights into its underlying mechanisms. In addition, quantified age-dependent repeat instability data may be useful when compared to age-dependent HD pathogenesis in investigating the temporal relationship between the two processes. However, progressive changes in *HTT* CAG repeat instability over an extensive period of time have not previously been quantified. We recently developed a method for quantifying instability from GeneMapper traces that can be used in various research applications [12] including quantitative assessment of the dynamics of repeat length changes. Here, as a part of our ongoing research efforts to understand mechanisms underlying CAG repeat instability and to understand better the role of CAG repeat instability in HD pathogenesis, we quantified repeat instability in tissues of *Hdh* CAG knock-in mice at various time points to determine how the *HTT* CAG repeat length changes over time.

## Results

**Figure 1:**
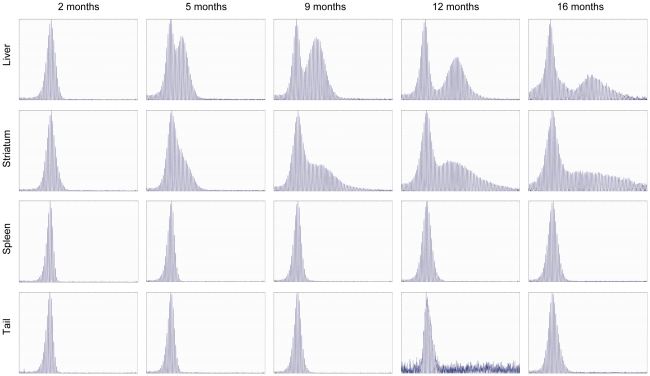
GeneMapper analysis of *Hdh^Q111^* CAG repeats shows pronounced age-dependent repeat instabilities in striatum and liver. Genomic DNA from liver, striatum, spleen and tail of *Hdh^Q111/+^* mice (CD1 background) was used for PCR amplification of the *HTT* CAG repeat followed by GeneMapper analysis. Five time points were used: 2 months (5 mice); 5 months (5 mice); 9 months (5 mice); 12 months (3 mice); 16 months (5 mice). Representative GeneMapper traces are shown.

We extracted genomic DNA from two unstable tissues (liver and striatum) and two stable tissues (spleen and tail) from *Hdh^Q111^* CAG knock-in mice (CD1 background; heterozygotes with 96–109 CAGs,) at various time points (2, 5, 9, 12, and 16 months) to quantify *HTT* CAG repeat instability. Similar to HD patients, these mice exhibit expansion-biased, time-dependent and tissue-specific *HTT* CAG repeat instability [8]. As shown in Figure 1, the *Hdh^Q111^* CAG repeats in spleen and tail were very stable, maintaining the patterns observed at 2 months at later time points. In contrast, the *Hdh^Q111^* CAG repeats in liver and striatum showed repeat instability at 5 months with levels of instability increasing over time. Interestingly, the pattern of repeat instability in liver differed from that of striatum. In liver, the population of unstable repeats remained distinct until 16 months, broadening slightly over time. In contrast, the shape of the initial distinctive population of unstable repeats in striatum broadened to a much greater degree over time, becoming diffuse at 16 months.

**Figure 2:**
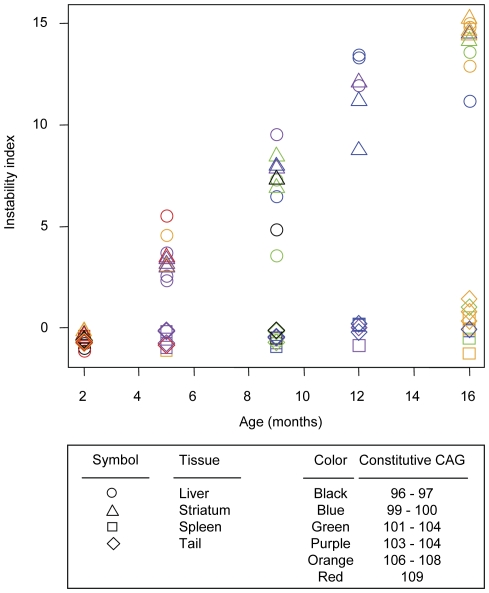
Age-dependent increase of somatic instability in liver and striatum suggests near linear increases in instability in these tissues. Somatic repeat instability was quantified from GeneMapper traces by determining an instability index (Methods) for striatum, liver, spleen and tail of each *Hdh^Q111^* ^/+^ mouse (CD1 background) at each time point: 2 months (5 mice); 5 months (5 mice); 9 months (5 mice); 12 months (3 mice); 16 months (5 mice). Instability index was plotted against age. Constitutive CAG repeat lengths are color-coded. Circle, liver; triangle, striatum; square, spleen; and diamond, tail.

Next, we quantified the levels of repeat instability using a recently developed method [12]. This method corrects background using a relative height threshold and normalizes data efficiently to generate an “instability index” that represents the mean CAG expansion size in a population of cells. As shown in Figure 2, instability indices of liver and striatum increased in an age-dependent manner, and the relationships between age and instability index appeared to be linear. However, instability indices in spleen and tail remained approximately the same over time, showing a slight increase in tail by 12–16 months. To calculate the rates of repeat length change and to test whether constitutive CAG repeat length significantly affects the instability index, we then modeled tissue instability index as a function of age, constitutive CAG repeat length, and their interaction. As summarized in Table 1, age was significantly associated with instability index in liver (1.04 CAG increase per month; standard error, 0.081), striatum (1.06 CAG increase per month; standard error, 0.03), and tail (0.09 CAG increase per month; standard error, 0.019), indicating that CAG repeats expand in these tissues in an age-dependent manner. Although age was significantly associated with instability index in tail, its rate of increase was 10-fold smaller than that of liver or striatum, suggesting a very small increase in instability in tail (Table 1). In addition, neither constitutive CAG repeat length nor the interaction between age and constitutive CAG repeat length was significantly associated with instability index in these tissues (Table 1). It has previously been shown that somatic instability is influenced by constitutive repeat length. For example, somatic instability was not observed in *Hdh* CAG knock-in mice with 48 CAG repeats [8]. Therefore, our data suggest that within the relatively narrow range of constitutive repeat lengths that we examined (96–109 CAGs), somatic expansion over a time frame of 16 months does not depend appreciably on constitutive repeat length.

**Table 1:** Statistical models of instability index by age and constitutive CAG length.

	Liver	Striatum	Spleen	Tail
Intercept(p-value)	0.0006539(0.999)	0.002079(0.989)	−0.001895(0.984)	0.007814(0.917)
Age(p-value)	1.0411626(7.92E-11)	1.061663(<2e-16)	0.026791(0.162)	0.0902(9.51E-6)
Constitutive CAG(p-value)	0.0997302(0.336)	0.019842(0.601)	−0.019192(0.414)	0.018469(0.340)
Age:constitutive CAG(p-value)	0.0005170(0.979)	0.001644(0.819)	−0.001498(0.735)	0.006179(0.101)

Instability index was modeled using age, constitutive CAG, and their interaction to understand rate of instability increase and to test effects of the constitutional repeat length on the rate of instability index change. Each variable was centered around the mean. Model parameters and p-values represent tissue-specific values.

**Figure 3:**
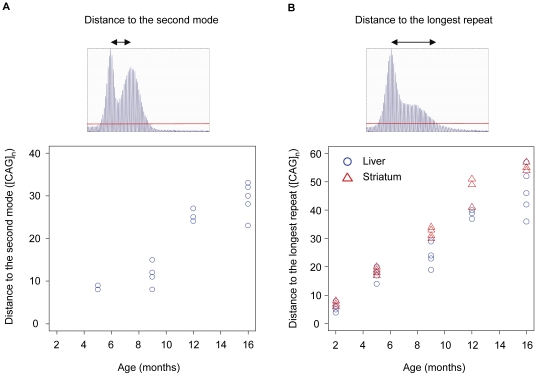
Quantification of the different patterns of somatic repeat instability in liver and striatum. To capture different patterns of repeat instability in liver and striatum, two additional measurements were made. (A) Repeats in liver showed a characteristic bimodal distribution and to quantify this, the distance between the modes of the constitutive and somatically expanded repeats was determined. The mode-mode distance was plotted at each time-point: 5 months (3 mice); 9 months (4 mice); 12 months (3 mice); 16 months (5 mice). All mice at 2 months, two mice at 5 months, and one mouse at 9 months did not show a distinct second mode. Two mice at 5 months showed the same distances between the two modes. (B) In striatum, the mode of the somatically expanded repeats was not distinct. Therefore, we measured the distance between the constitutive repeat mode and the longest repeat after background correction, and compared these to those of liver at each time point: 2 months (5 mice); 5 months (5 mice); 9 months (5 mice); 12 months (3 mice); 16 months (5 mice). Blue circle, liver; red triangle, striatum. Red line represents relative peak threshold based on 10% threshold factor that was used for background correction.

It is also interesting to note that liver and striatum showed similar instability indices and that the rates of instability index increase were not significantly different from each other (p-value 0.669) even though the patterns of repeat instability in these tissues were quite different (Figure 1). As the instability index measures mean repeat size changes from the constitutive repeat length, it is not well suited for comparing qualitatively different patterns of repeat instabilities. Therefore, we made additional measurements to characterize the qualitatively different patterns of repeat instability in liver and striatum. Repeats in liver showed two discrete populations, representing a bimodal distribution comprising CAG repeats centered around the constitutive repeat (i.e., stable repeats) and those further expanded (i.e., unstable repeats). Since the unstable repeat population in liver migrated to the right over time while maintaining its shape, we measured the distance between the two modes to capture this characteristic repeat instability in liver. As shown in Figure 3A, the distance between the two modes in liver increased in an age-dependent manner, and the mode of expanded repeats migrated to the right at a rate of ∼2.1 CAG units per month (linear regression: standard error 0.248; p-value 1.29×10^−6^). This suggested that once CAG repeats in liver expand the same population of repeats gains approximately 2 CAG units per month repeatedly. In striatum, the bimodal distribution of repeats was much less obvious with the population of unstable repeats being more widely distributed. To capture this characteristic pattern of repeat instability in striatum, we measured the distance between the constitutive repeat mode and the longest repeat after background correction [12]. As shown in Figure 3B, the distance between the constitutive repeat and the longest repeat increased over time in both striatum (∼3.5 CAG increase per month; standard error, 0.1095; p-value<2×10^−16^) and liver (∼2.9 CAG increase per month; standard error, 0.2007; p-value, 2.13×10^−12^). However, striatum showed a significantly increased rate of change in distance compared to that of liver (p-value, 0.00764), implying that units of additional CAG repeats obtained by striatum are more broadly distributed and more dynamic than those in liver.

**Figure 4:**
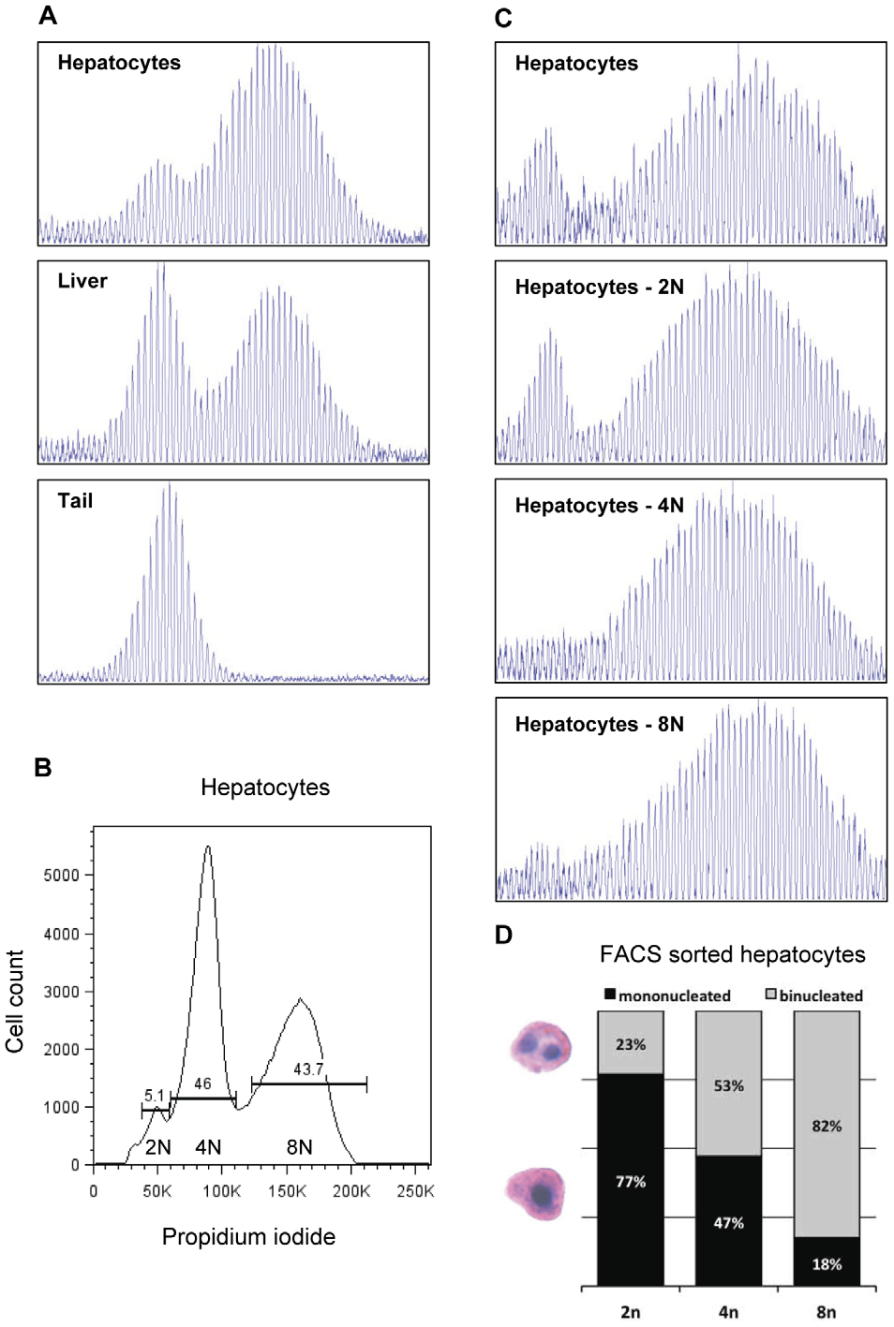
Hepatocytes are enriched for unstable *HTT* CAG repeats particularly when in polyploidy states. (A) GeneMapper traces of PCR-amplified *HTT* CAG repeats from isolated hepatocytes (top panel), whole liver (middle panel) and tail DNA (bottom panel) from a 4 month old *Hdh^Q111/+^* mouse (FVB/N background, constitutive CAG142) were shown. Isolated hepatocytes show an enrichment of unstable CAG repeats when compared to a whole liver preparation. (B, C and D) Hepatocytes isolated from a 9 month old *Hdh^Q111/+^* mouse (C57BL/6J background, constitutive CAG 131) were fixed, stained with propidium iodide and FACS sorted based on DNA content into 2N, 4N and 8N hepatocytes pools. (B) FACS analysis indicated that approximately 95% of the hepatocytes were in polyploid states (4N or 8N). X-axis and Y-axis represent DNA content and cell count, respectively. (C) GeneMapper traces of PCR-amplified *HTT* CAG repeat of unsorted hepatocytes (top panel: mixture of 2N, 4N and 8N hepatocytes) and FACS sorted 2N, 4N and 8N hepatocytes pools revealed that stable CAG repeats are almost exclusively present in the 2N hepatocytes pool and that unstable CAG repeats are strongly enriched in both 4N and 8N hepatocytes pools. (D) Cytological analysis of the FACS sorted hepatocytes confirmed a decline of mononuclearity with increasing nuclear content [19]. However, due to the lack of a clear boundary between 2N and 4N cells, the FACS sorted 2N hepatocytes pool was susceptible to contamination from the 4N cells, resulting in the presence of a significant proportion of binucleated hepatocytes (23%) in the 2N hepatocyte pool. It is also important to note that mononucleated 2N pools may contain mononucleated 4N cells.

The relatively well-defined population of unstable repeats in liver compared to striatum could be the result of different mechanisms of repeat expansion in the two tissues and/or a more heterogeneous population of unstable cells in striatum than in liver. Previous studies have shown that the *HTT* CAG repeat is unstable in both neurons and glia with neurons showing more instability than glia [16], [17]. However, cell-type specificity for *HTT* CAG instability in liver has not previously been investigated, and therefore we examined whether the defined population of unstable repeats in liver might be enriched in a specific cell-type. To test this we isolated hepatocytes from an *Hdh^Q111^* ^/+^ mouse and measured *HTT* CAG repeat lengths in comparison to whole liver. Interestingly, the unstable repeats were considerably enriched relative to the stable repeats in the isolated hepatocyte preparation (Figure 4A).

We were interested in investigating in further detail the origin of the stable and unstable repeats in hepatocytes. Hepatocytes undergo a polyploidization process, beginning at weaning, and by adulthood ∼95% of hepatocytes in the mouse are either 4N tetraploid (mononucleated or binucleated) or 8N octaploid (mononucleated or binucleated) [18], [19]. We therefore sought to isolate polyploid hepatocytes as a means of obtaining cell populations representing the vast majority of hepatocytes. Fluorescence activated cell sorting (FACS) analysis of a hepatocyte preparation from a second *Hdh^Q111^* ^/+^ mouse revealed ∼5%, 46% and 44% of 2N, 4N and 8N hepatocytes, respectively (Figure S1; Figure 4B), and morphological assessment confirmed the absence of non-hepatocytes in these pools of cells. Subsequent analysis of *HTT* CAG repeat lengths of FACS sorted cells showed that 4N and 8N hepatocytes contained almost exclusively the expanded repeat population (Figure 4C). Interestingly, the 2N hepatocytes contained both stable and unstable repeats (Figure 4C). Due to the relatively small proportion of 2N hepatocytes (5.1% of all hepatocytes; Figure 4B), obtaining enough highly pure 2N hepatocytes was technically challenging, resulting in the presence of 23% binucleated hepatocytes in 2N pool (Figure 4D). These results suggested that unstable repeats in the 2N pool may be contributed by 4N cell contamination in the FACS sorted 2N pool. Based on these observations, we predicted that the percentage of DNA from 4N cells in DNA of the 2N pool may be as much as 60% (Figure S2A). In addition, in mixing experiments, we observed that when 25% of tail DNA (stable repeats) was mixed with 75% of 8N hepatocytes DNA (unstable repeats), the distribution of repeats (Figure S2B; 25∶75) resembled that of isolated 2N cells (Figure S2B, top panel), suggesting that contaminating polyploid hepatocytes are likely to contribute to some of the unstable peaks in the sorted 2N hepatocyte pool (Figure S2B). Thus, while we are confident that stable repeats are present in 2N hepatocytes and unstable repeats in the 2N pool are likely contributed by 4N hepatocytes, we cannot unequivocally determine whether the 2N hepatocytes contain *exclusively* stable repeats due to technical difficulties in obtaining highly pure 2N hepatocytes. Therefore, our data demonstrate that hepatocytes contain at least the vast majority of the expanded *HTT* CAG repeats in the liver, and support that the expansions may preferentially arise in hepatocytes that are in a polyploid state.

## Discussion

By quantifying *HTT* CAG repeat instability in tissues at multiple time points, we show that repeat instability in liver and striatum of *Hdh^Q111^* mice increases over time, supporting published qualitative data indicating that repeat expansion is a recurring process [8], [9], [10], [16]. Our data are consistent with a mechanism in which somatic expansions accumulate by incremental small repeat length changes. Based on our results (see Figure S3 for a graphical representation), we propose that the *Hdh^Q111^* CAG repeats in liver and striatum expand continuously, with similar overall levels of repeat expansions in these tissues; on average 1 CAG unit increase per month. However, the distributions of unstable repeats in these tissues are different. The unstable repeats in liver gain similar sizes of additional repeats over time (approximately 2 CAG units per month), maintaining a well-defined population of unstable repeats. In contrast, the unstable repeat population in striatum gains broadly distributed sizes of additional repeats, and the longest repeat lengthens at a rate of approximately 3.5 CAG units per month per cell resulting in a less defined population of expanded repeats. While this is the simplest interpretation of the data, given the nature of our cross-sectional study in which instability is examined at 3–4 month intervals, it is also plausible that CAG increases of greater magnitude could occur less frequently. A denser cross-sectional time-course, or ideally a longitudinal analysis that may be feasible in liver, would allow greater resolution of the repeat length changes that occur over time.

In contrast to the continuous expansions of unstable repeats, we did not observe any significant shift or migration of the constitutive repeat over time in liver or striatum (data not shown). While analysis of GeneMapper traces does not readily permit quantification of the relative levels of constitutive versus somatically expanded repeat lengths, these data are consistent with the presence of stable and unstable populations of repeats in both liver and striatum at 16 months of age.

Notably, consistent with a number of studies in HD mice [9], [16], [20] we did not observe the periodicity in the instability profile in striatum that was recently described in the R6/1 exon 1 HD mouse model (CBA/B6J background) [21], and interpreted to be the result of successive insertions of an average of 7 CAGs. The different GeneScan/GeneMapper profiles may reflect differences in the genomic location of the repeat, CAG repeat length or genetic background as well as the precise experimental conditions. Although we have not observed such periodic expansion in the striatum in a number of genetic backgrounds (Figure S4), it is clear that genetic background does influence striatal instability [22]. Possible candidate genes include DNA repair genes known to play a role in *HTT* CAG repeat instability [13], [14], [20], [23].

The increases in instability index in liver and striatum that we observed appeared to be linear over the relatively narrow range of constitutive repeat lengths of 96–109 CAGs, suggesting that within this repeat length range the gain of additional CAG repeats does not dramatically accelerate the rate of repeat instability within a relatively short period of time. However, it may be still possible to see accelerated rates of repeat expansion if instability were measured at later time points in these mice. In HD patients, where the repeat can expand over decades from ∼40 CAGs to hundreds of CAGs [7], it is very possible that somatic expansion accelerates over time, consistent with dramatic differences in instability in *Hdh* CAG knock-in mice bearing repeat lengths from 48 to 109 repeats [8].

The phenomenon of similar overall repeat instabilities with different patterns of unstable repeats in liver and striatum is quite intriguing. Migration of the unstable repeats to the right in the liver, whilst maintaining a similar distribution about the mode, supports the notion that similar sizes of repeats are successively added in liver. In contrast, striatum showed a distinct population of unstable repeats initially, but the shape of the unstable repeat population became broader as the mice aged, generating a distribution of unstable repeats that resembled a uniform distribution at 16 months. We determined that the unstable repeats are highly enriched in polyploid hepatocytes, suggesting that the relatively tight distribution of unstable repeats in the liver may be due to their occurrence in a unique cell population. We were not able to determine unequivocally whether the unstable alleles were associated *solely* with the polylpoid hepatocytes, however our data indicate that 2N hepatocytes do contain stable repeats, suggesting that the polyploid cells reflect a state that is permissive to repeat expansion. These data support previous findings in a mouse model of Myotonic Dystrophy Type 1 (DM1) in which expanded *DM1* CTG alleles were associated with polyploid hepatocytes [24], suggesting that similar mechanisms underlie the instability of these two disease-associated repeats in the liver. Polyploidy is associated with a state of terminal differentiation of hepatocytes and by adulthood most (95%) hepatocytes are polyploid. The development of prominent repeat instability after 2 months of age (Figure 1) is therefore consistent with expansions arising in non-dividing cells, as previously predicted [12].

Interestingly, a prominent bimodal repeat length distribution is apparent in liver in some [7], [21], [25] but not all HD mouse models examined [9]. Genetic background, *cis* sequences or CAG repeat length may account for the difference, as discussed above. Notably, we have observed a bimodal distribution of repeat lengths in *Hdh^Q111^* ^/+^ mice in five genetic backgrounds - CD1, C57BL/6J, C57BL/6N, FVB/N, 129S2/SvPasCrlf (Figures 1, 4 and Figure S4), indicating that, at least qualitatively, similar liver instability can occur across a number of backgrounds when controlling for other factors. It may be that liver instability is relatively sensitive to environmental differences, e.g., diet that could alter metabolism. Indeed, we have previously found that liver instability correlates with expression of metabolism genes [12]. Interestingly, hepatocyte polyploidy is controlled by insulin concentration [26], suggesting that insulin levels might modify instability in the liver.

CAG repeat instability in target tissues such as the striatum has been hypothesized to contribute to HD pathogenesis in *Hdh^Q111^* mice and in patients [13], [14], [15]. Our quantitative analyses indicate that the dynamics of *HTT* CAG expansion in two highly unstable tissues, striatum and liver, are different. Different mechanisms of expansion and/or cell types may account for this, the latter indicated by the enrichment of expanded alleles in polyploid hepatocytes. Different factors are predicted to contribute to liver and striatal instability [12], and understanding these factors will be critical in finding ways of preventing somatic expansion in the striatum. The findings reported here will contribute to expanding our knowledge of repeat instability in terms of its underlying mechanisms and involvement in disease.

## Materials and Methods

### Mice

*Hdh^Q111/+^* knock-in mice (CD1 background; heterozygote; mixed gender; 5 mice for 2, 5, 9 and 16 months; 3 mice for 12 months) [27] were used for cross-sectional quantification of tissue instability in liver, striatum, spleen and tail over time. The CAG repeat lengths of these mice were 96 (1), 97 (2), 99 (2), 100 (2), 101 (2), 102 (1), 103 (3), 104 (2), 106 (1), 108 (5), and 109 (2). The range, mean, and standard deviation of the constitutive CAG repeat length was 96–109, 103, and 4.19, respectively. For a given time point, mice comprised sets of littermates, and tissues were harvested together. *Hdh^Q111^* ^/+^ mice on FVB/N [22] and C57BL/6J backgrounds were used for hepatocyte isolation experiments. Additional *Hdh^Q111^* ^/+^ mice on C57BL/6J, C57BL/6N and 129S2/SvPasCrlf backgrounds [22] were used to determine liver instability. Details of all the mice used are displayed in Table S1. C57BL/6J mice were generated from the *Hdh^Q111^* C57BL/6N congenic line [22] by a speed congenics backcross strategy using a 768-SNP panel containing 19 SNPs that distinguish C57BL/6J from C57BL/6N strains [28] for three rounds of breeding followed by five standard backcross generations. All animal experiments were performed to minimize pain and discomfort, under an approved protocol of the Massachusetts General Hospital Subcommittee on Research Animal Care (SRAC; protocol number, 2009N000216).

### Instability quantification

Each step of the experimental procedure (e.g., DNA extraction, PCR and GeneMapper analysis) was performed at a same time for a given time point. Genomic DNA (300 ng), isolated from mouse tissues (DNeasy, Qiagen), was used for PCR amplification using *HTT* CAG repeat-specific primers as previously described [13]. PCR products were separated using the ABI 3730 DNA analyzer (Applied Biosystems), and signals were processed using GeneMapper v.3.7 software. To assign correct repeat size, GeneScan 500-LIZ as internal size standard was added in each analysis. GeneMapper traces were used to determine an instability index as described [12]. Briefly, the highest peak in each analysis was identified (main allele), and 10% (threshold factor) of the height of the highest peak was set as a relative peak height threshold. For background correction, peaks with height less than the threshold were excluded, and normalized peak heights were calculated by dividing the peak height of each peak by the sum of the heights of all signal peaks. Then, the change in CAG length of each peak was calculated from the highest peak in each analysis (main allele). Finally, the normalized peak height was multiplied by the change from the main allele, and these values were summed to get the instability index, which represents the mean CAG repeat length change in the population of cells being analyzed.

### Isolation of hepatocytes and FACS sorting based on DNA content

Hepatocytes were isolated from liver as previously described by van den Broek *et al.* [24]. In essence, mice were terminally anaesthetized with tribromoethanol (0.5 mg/g), followed by left ventricular perfusion with: 30 ml heparinized PBS (2 U/ml); 30 ml HEPES buffer (25 mM) with 0.25 mM EGTA; 10 ml HEPES buffer; and finally 25 ml HEPES buffer containing 2 mM CaCl_2_ and 0.025% (w/v) collagenase IV (Gibco). Following dissection of the liver, cells were gently filtered through a 70 µm nylon cell strainer (BD Falcon). Hepatocyte enrichment was performed by three sequential washes with DMEM complete media (DMEM, 10% FBS, 400 µg/ml gentamycin, 100 IU/ml penicillin, 100 µg/ml streptomycin, 2 mM L-Glutamine) in combination with low speed centrifugation at 500 rpm for five minutes. The cells were subsequently fixed in 70% ethanol overnight at −20°C. Following a wash with PBS, the cells were stained in PBS containing 20 µg/ml propidium iodide and 1 mg/ml RNase, for 15 minutes at room temperature. Finally, hepatocytes were FACS sorted into 2N, 4N and 8N populations using a BD 5 laser SORP FACS Vantage SE Diva system (MGH Flow Cytometry Core facility). Cytological analysis of FACS sorted cells (approximately 100 cells) was performed by standard hematoxylin and eosin (H&E) staining.

### Statistical analysis

To test whether age, constitutive CAG repeat length, or their interaction contributed to the instability index, multiple regression models were fitted to data for each tissue. All variables were centered around the corresponding mean value. All statistical analysis was done using R (version, 2.7.2).

## Supporting Information

Figure S1 **FACS analysis of mouse hepatocytes based on DNA content.** Hepatocytes isolated from a 9 month old *Hdh^Q111/+^* mouse (C57BL/6J, constitutive CAG 131) were fixed, stained with propidium iodide and FACS sorted based on DNA content into 2N, 4N and 8N hepatocyte pools. (A) FACS light scatter plot representing the population of cells selected based on size and granularity, as indicated by purple box. (B) Selected cells were further gated by propidium iodide signal. The purple box represents the final population of cells that was FACS sorted according to nuclear content, which contained 100% hepatocytes judged by morphology of cells (H&E staining).
(PDF)

Figure S2 **Assessment of the extent to which the unstable repeats detected in the 2N hepatocytes pool is due to contamination from 4N cells.** (A) In order to calculate the levels of polyploidy contamination in 2N hepatocytes pool, we hypothesized that binucleated cells in the 2N pool are contamination from binucleated 4N cells. All calculations were based on 100 cells. FACS sorted cells were examined by H&E staining for cell morphology and nuclearity (Figure 4D), and all FACS sorted cells were 100% hepatocytes. ^$^ Since 23 binucleated 2N cells likely originated from the binucleated 4N cell population, one can assume that a pool of 77 mononucleated 2N cells contains approximately 20 mononucleated 4N cells considering the 47∶53 ratio of mono∶binucleated 4N cells. Therefore, 100 FACS sorted 2N cells were predicted to comprise 57 mononucleated 2N, 20 mononucleated 4N and 23 binucleated 4N cells. Based on this composition, we predicted that approximately 60% of the DNA from 2N hepatocytes pool is actually contributed by 4N hepatocytes. (B) To assess whether the signal from the expanded repeats in 2N pool (top panel) might arise from unstable alleles present in contaminating polyploid hepatocytes we conducted a mixing experiment whereby we mixed DNA from tail (containing only stable repeats) with DNA from 8N cells (containing mostly expanded alleles) in various proportions (bottom panels). Based on the traces obtained with proportions of 50∶50 & 25∶75 stable∶unstable repeats, and our calculation of 60% contaminating DNA, it appears that the majority of the unstable repeats in the 2N pool GeneMapper trace is likely to arise from unstable repeats that originate in contaminating polyploid hepatocytes.
(PDF)

Figure S3 **Graphical illustration of age-dependent instability in liver and striatum.** To illustrate age-dependent repeat instability in liver and striatum, outlines of representative GeneMapper traces were drawn. (A) We propose that unstable repeats in liver recurrently gain similar sizes of additional repeats, resulting in migration of the unstable repeat population to the right as the mice age. (B) In contrast, we propose that unstable repeats in striatum gain different sizes of additional repeats, leading to broadening of the initial distinctive unstable repeat population. Arrow represents the constitutive repeat.
(PDF)

Figure S4 ***Hdh^Q111/+^*** **mice on different genetic backgrounds show qualitatively similar bimodal distributions of repeats in liver.** GeneMapper traces of PCR-amplified *HTT* CAG repeat from liver DNA of *Hdh^Q111^* ^/+^ mice with different genetic backgrounds (C57BL/6J, C57BL/6N, 129S2/SvPasCrlf). Mice are congenic on these backgrounds as determined by SNP typing. Constitutive CAG repeat numbers and ages of the mice are shown on top of each panel.
(PDF)

Table S1 **Mice used in this study.**
(DOC)
