# Supplementary figures and images for: Quantification of Age-Dependent Somatic CAG Repeat Instability in Hdh CAG Knock-In Mice Reveals Different Expansion Dynamics in Striatum and Liver
Source: PLoS One. 2011 Aug 29;6(8):e23647. doi: 10.1371/journal.pone.0023647 (PMC3163641; doi:10.1371/journal.pone.0023647)

**A**

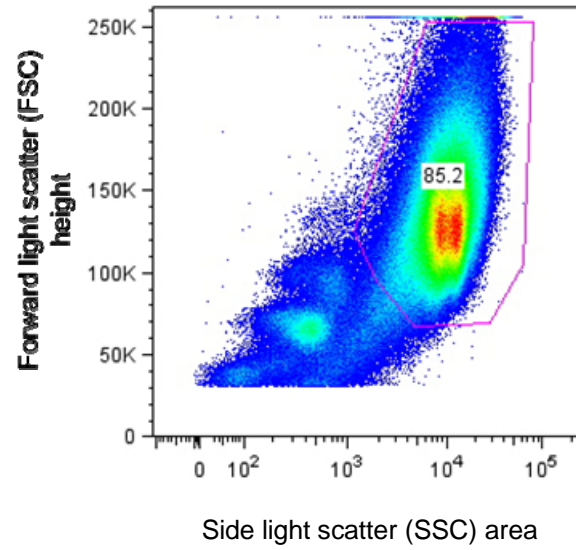

**B**

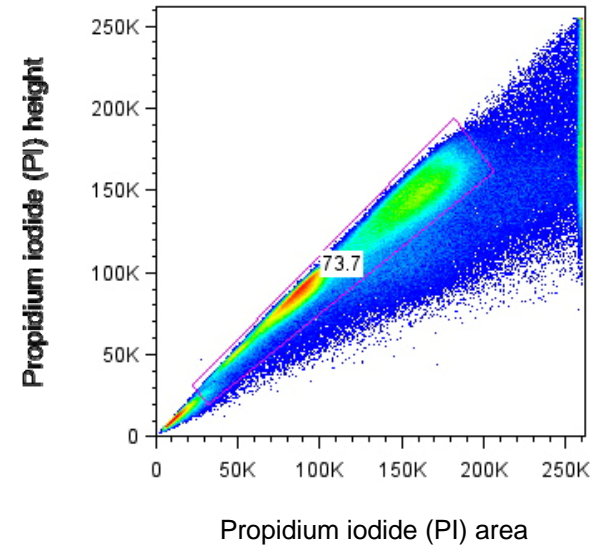

Supplement: Figure S1 — FACS analysis of mouse hepatocytes based on DNA content. Hepatocytes isolated from a 9 month old HdhQ111/+ mouse (C57BL/6J, constitutive CAG 131) were fixed, stained with propidium iodide and FACS sorted based on DNA content into 2N, 4N and 8N hepatocyte pools. (A) FACS light scatter plot representing the population of cells selected based on size and granularity, as indicated by purple box. (B) Selected cells were further gated by propidium iodide signal. The purple box represents the final population of cells that was FACS sorted according to nuclear content, which contained 100% hepatocytes judged by morphology of cells (H&E staining). (PDF) [file pone.0023647.s001.pdf]

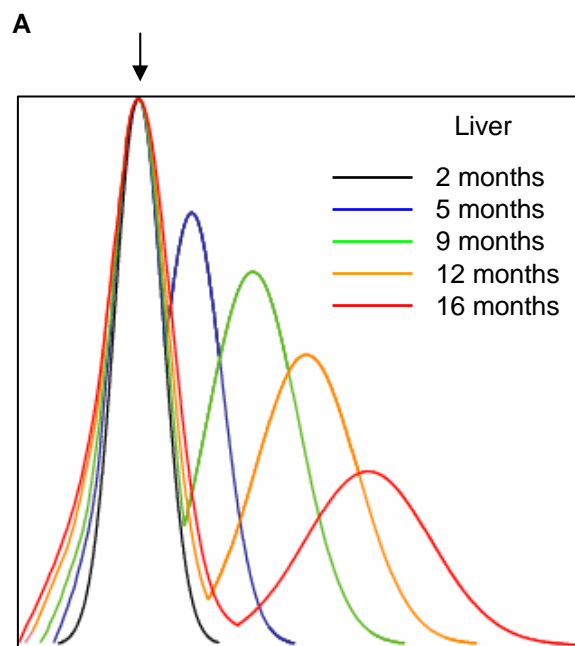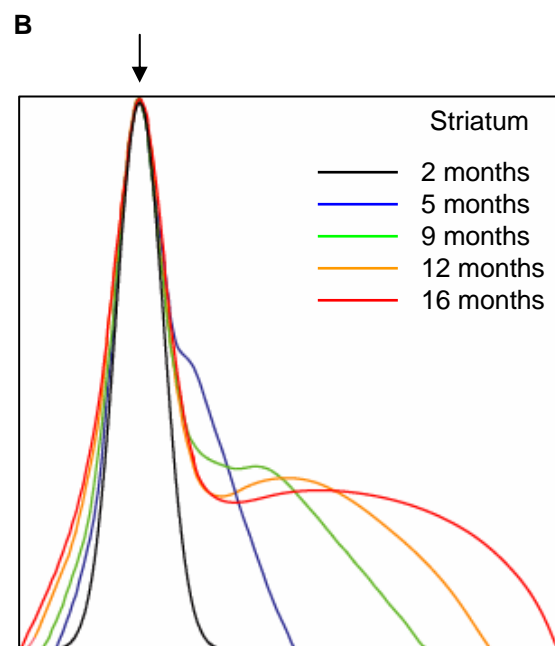

Supplement: Figure S3 — Graphical illustration of age-dependent instability in liver and striatum. To illustrate age-dependent repeat instability in liver and striatum, outlines of representative GeneMapper traces were drawn. (A) We propose that unstable repeats in liver recurrently gain similar sizes of additional repeats, resulting in migration of the unstable repeat population to the right as the mice age. (B) In contrast, we propose that unstable repeats in striatum gain different sizes of additional repeats, leading to broadening of the initial distinctive unstable repeat population. Arrow represents the constitutive repeat. (PDF) [file pone.0023647.s003.pdf]

Background  
Age (months)  
CAG

C57BL/6J  
9  
128

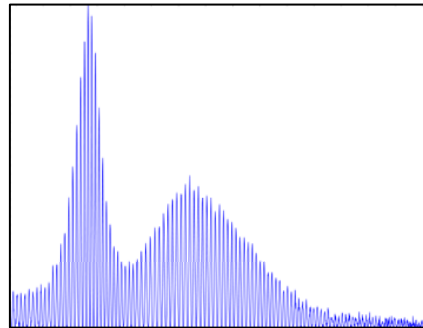

C57BL/6N  
6  
114

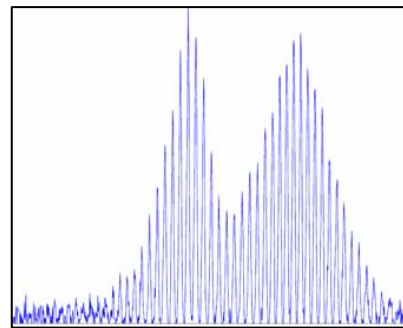

129S2/SvPasCrlf  
6  
108

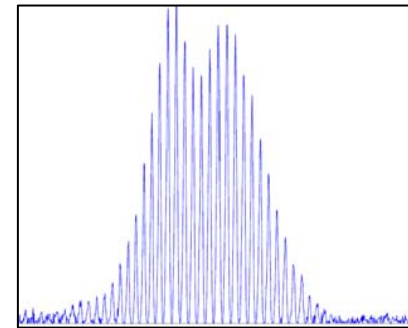

Supplement: Figure S4 — HdhQ111/+ mice on different genetic backgrounds show qualitatively similar bimodal distributions of repeats in liver. GeneMapper traces of PCR-amplified HTT CAG repeat from liver DNA of HdhQ111 /+ mice with different genetic backgrounds (C57BL/6J, C57BL/6N, 129S2/SvPasCrlf). Mice are congenic on these backgrounds as determined by SNP typing. Constitutive CAG repeat numbers and ages of the mice are shown on top of each panel. (PDF) [file pone.0023647.s004.pdf]
