# Supplementary material for: Quantification of Age-Dependent Somatic CAG Repeat Instability in Hdh CAG Knock-In Mice Reveals Different Expansion Dynamics in Striatum and Liver
Source: PLoS One. 2011 Aug 29;6(8):e23647. doi: 10.1371/journal.pone.0023647 (PMC3163641; doi:10.1371/journal.pone.0023647)

A

Predicted contamination of 4N cells in 2N hepatocytes

	2N		4N	
	mono	bi	mono	bi
Observed numbers of mononucleated and binucleated hepatocytes in FACS sorted cells (/ 100 cells)	77	23	47	53
$ Predicted numbers of mononucleated and binucleated hepatocytes in FACS sorted 2N cells (/ 100 cells)	57	0	20	23
Predicted percentages of DNA of 2N and 4N cells in DNA from 2N pool	40%		60%	

B

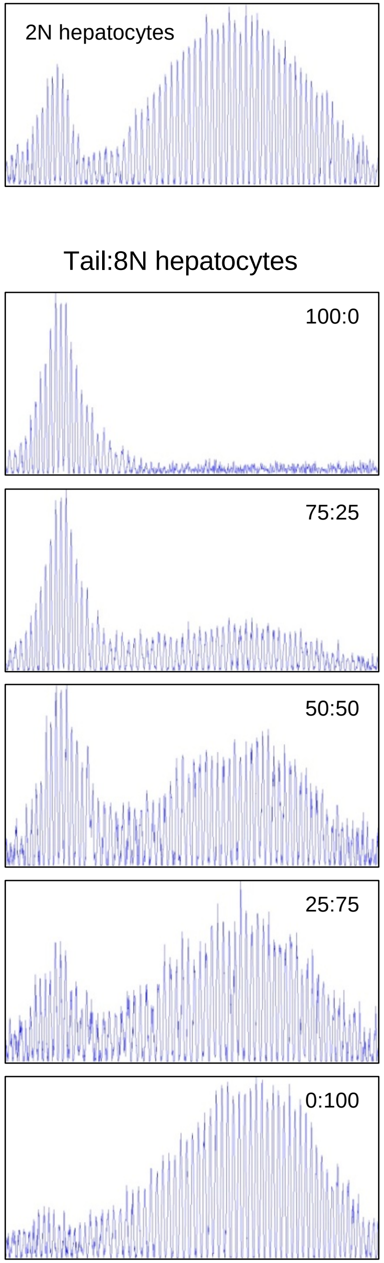

Supplement: Figure S2 — Assessment of the extent to which the unstable repeats detected in the 2N hepatocytes pool is due to contamination from 4N cells. (A) In order to calculate the levels of polyploidy contamination in 2N hepatocytes pool, we hypothesized that binucleated cells in the 2N pool are contamination from binucleated 4N cells. All calculations were based on 100 cells. FACS sorted cells were examined by H&E staining for cell morphology and nuclearity (Figure 4D), and all FACS sorted cells were 100% hepatocytes. $ Since 23 binucleated 2N cells likely originated from the binucleated 4N cell population, one can assume that a pool of 77 mononucleated 2N cells contains approximately 20 mononucleated 4N cells considering the 47∶53 ratio of mono∶binucleated 4N cells. Therefore, 100 FACS sorted 2N cells were predicted to comprise 57 mononucleated 2N, 20 mononucleated 4N and 23 binucleated 4N cells. Based on this composition, we predicted that approximately 60% of the DNA from 2N hepatocytes pool is actually contributed by 4N hepatocytes. (B) To assess whether the signal from the expanded repeats in 2N pool (top panel) might arise from unstable alleles present in contaminating polyploid hepatocytes we conducted a mixing experiment whereby we mixed DNA from tail (containing only stable repeats) with DNA from 8N cells (containing mostly expanded alleles) in various proportions (bottom panels). Based on the traces obtained with proportions of 50∶50 & 25∶75 stable∶unstable repeats, and our calculation of 60% contaminating DNA, it appears that the majority of the unstable repeats in the 2N pool GeneMapper trace is likely to arise from unstable repeats that originate in contaminating polyploid hepatocytes. (PDF) [file pone.0023647.s002.pdf]
