# Supplementary material for: Quantification of Age-Dependent Somatic CAG Repeat Instability in Hdh CAG Knock-In Mice Reveals Different Expansion Dynamics in Striatum and Liver
Source: PLoS One. 2011 Aug 29;6(8):e23647. doi: 10.1371/journal.pone.0023647 (PMC3163641; doi:10.1371/journal.pone.0023647)
Supplement: Table S1 — Mice used in this study. (DOC) [file pone.0023647.s005.doc]

**Table S1. Mice used in this study.**

| Mouse number | Gender | Constitutive repeat length | Time point (month) | Genetic background |
| --- | --- | --- | --- | --- |
| 1 | M | 97 | 2 | CD1 |
| 2 | M | 97 | 2 | CD1 |
| 3 | M | 108 | 2 | CD1 |
| 4 | M | 108 | 2 | CD1 |
| 5 | M | 109 | 2 | CD1 |
| 6 | F | 108 | 5 | CD1 |
| 7 | F | 104 | 5 | CD1 |
| 8 | F | 109 | 5 | CD1 |
| 9 | M | 103 | 5 | CD1 |
| 10 | M | 103 | 5 | CD1 |
| 11 | M | 101 | 9 | CD1 |
| 12 | F | 101 | 9 | CD1 |
| 13 | F | 103 | 9 | CD1 |
| 14 | F | 99 | 9 | CD1 |
| 15 | F | 96 | 9 | CD1 |
| 16 | F | 100 | 12 | CD1 |
| 17 | F | 99 | 12 | CD1 |
| 18 | F | 104 | 12 | CD1 |
| 19 | M | 100 | 16 | CD1 |
| 20 | M | 108 | 16 | CD1 |
| 21 | F | 102 | 16 | CD1 |
| 22 | F | 108 | 16 | CD1 |
| 23 | F | 106 | 16 | CD1 |
| 24 | M | 142 | 4 | FVB/N |
| 25 | M | 131 | 9 | C57BL/6J |
| 26 | F | 128 | 9 | C57BL/6J |
| 27 | F | 114 | 6 | C57BL/6N |
| 28 | F | 108 | 6 | 129S2/SvPasCrlf |

Mice 1-23 were used in the cross-sectional study (Figure 1), mice 24 and 25 in the hepatocyte isolation study (Figure 4, Figure S2) and data from mice 26-28 are shown in Figure S4.

Constitutive repeat length of each mouse was determined from the GeneMapper analysis of tail.
